# Modulation of Osteoclastogenesis with Macrophage M1- and M2-Inducing Stimuli

**DOI:** 10.1371/journal.pone.0104498

**Published:** 2014-08-07

**Authors:** Sujeeve Jeganathan, Cara Fiorino, Urja Naik, He song Sun, Rene E. Harrison

**Affiliations:** 1 Ontario Cancer Institute and Princess Margaret Cancer Centre, University Health Network, Toronto, Ontario, Canada; 2 Department of Biological Sciences, University of Toronto Scarborough, Toronto, Ontario, Canada; Tulane University, United States of America

## Abstract

Macrophages are generated through the differentiation of monocytes in tissues and they have important functions in innate and adaptive immunity. In addition to their roles as phagocytes, macrophages can be further differentiated, in the presence of receptor activator of nuclear factor kappa-B ligand (RANKL) and macrophage colony-stimulating factor (M-CSF), into osteoclasts (multinucleated giant cells that are responsible for bone resorption). In this work, we set out to characterize whether various inflammatory stimuli, known to induce macrophage polarization, can alter the type of multinucleated giant cell obtained from RANKL differentiation. Following a four-day differentiation protocol, along with lipopolysaccharide (LPS)/interferon gamma (IFNγ) as one stimulus, and interleukin-4 (IL-4) as the other, three types of multinucleated cells were generated. Using various microscopy techniques (bright field, epifluorescence and scanning electron), functional assays, and western blotting for osteoclast markers, we found that, as expected, RANKL treatment alone resulted in osteoclasts, whereas the addition of LPS/IFNγ to RANKL pre-treated macrophages generated Langhans-type giant cells, while IL-4 led to giant cells resembling foreign body giant cells with osteoclast-like characteristics. Finally, to gain insight into the modulation of osteoclastogenesis, we characterized the formation and morphology of RANKL and LPS/IFNγ-induced multinucleated giant cells.

## Introduction

Macrophages, produced by the differentiation of monocytes in tissues, play essential roles in non-specific disease defense (innate immunity) and in the initiation of specific defense mechanisms (adaptive immunity) [1]. Macrophages, along with neutrophils, dendritic cells, mast cells and monocytes, are termed professional phagocytes for their abilities to detect, destroy or sequester most foreign objects, infectious pathogens, and cancer cells.

One of the unique abilities of macrophages is to fuse with other macrophages to form multinucleated giant cells (MGCs) [2], [3]. MGCs are commonly found in the human body, with some examples being osteoclasts and foreign body giant cells. In addition, some MGCs, such as Langhans giant cells, and Toutons giant cells, are found in disease states or found associated with certain tumours (Giant Cell Tumours of the Bone) [2], [3], [4],[5]. In order to study these various MGCs, it is important to understand their formation and function.

The most characterized type of MGC is the osteoclast (OC). OCs have been generated *in vitro* and *in vivo* using rodent and human monocytes and macrophages. A key breakthrough in OC biology was the identification of macrophage colony stimulating factor (M-CSF) and receptor activator of nuclear factor kappa-B ligand (RANKL) as key molecules that will induce the differentiation of monocytes and macrophages into OCs [6]. Additionally, studies performed on RAW 264.7 murine macrophages showed that these cells respond to RANKL stimulation alone (without the need for M-CSF) *in vitro* to generate functional OCs, permitting OC research without the need of primary precursors [7]. Since these observations, other stimuli, such as bacterial lipopolysaccharides (LPS) [8], and interleukins (ILs) [9], [10] have also been shown to both promote and inhibit OC formation.

A challenge with MGCs and their formation is their *in vitro* characterization. There are numerous contradictory data, partly due to the manner by which they are generated and also the tools used to assess them. As a consequence, proper characterization of their features (biomarkers, functional assays) becomes difficult. We chose to perform our experiments on non-bone substrates. OCs, whether they are directly collected from humans and animals or generated from precursor primary cells or cell lines, have been shown to share similar characteristics when plated on bone or non-bone substrates [6], [7], [11], [12]. In addition to their bone resorption abilities, they show MMP-9 [13], [14] and cathepsin K (CK) [13], protein expression, and are TRAP-positive [13]. Additionally, most studies report that OCs plated on non-bone substrates are incapable or very weakly capable of some phagocytic activity [14].

The concept of macrophage polarization has been well studied in immunology. Polarized macrophages can be classified in two main groups: classically activated macrophages (or M1) and alternatively activated macrophages (or M2). M1 macrophages are generated by IFNγ and LPS whereas M2 macrophages can be generated by exposure to IL-4 or IL-13 (to yield M2a macrophages), immune complexes in combination with IL-1β or LPS (to yield M2b macrophages) or IL-10, TGFβ or glucocorticoids (to yield M2c macrophages) [15]. M1 macrophages are effective at host defense and clearing pathogens, while M2 macrophages are important for resolution of inflammation and tissue repair [16]. The classical M1 and M2 activation phenotypes represent two ends of a spectrum of macrophage polarization states that are induced by multiple factors and are characterized by expression of proteins that underlie specialized functions.

In this study, we investigated what happens when two distinct functions of macrophages – fusion and polarization – converge *in vitro*. Clinically, bacterial, viral and fungal infections of bones and joints can lead to various diseases such as osteomyelitis, reactive arthritis and septic arthritis [17], [18], [19]. The trigger in these cases is the formation and accumulation of MGCs to combat the invading pathogen. In order to fully understand the effects of M1- and M2-inducing agents on OC formation, we first committed RAW 264.7 murine macrophages towards the osteoclast lineage by chronically incubating them with RANKL. We then stimulated these cells with classically activating and alternatively activating agents (LPS/IFNγ and IL-4, respectively). Here we report that RANKL treatment of macrophages does not commit them to become OCs when they are subjected to LPS/IFNγ or IL-4 treatment. Instead, two other forms of MGCs that are known to have specific immunological roles are generated. We characterized these MGCs and also investigated the mechanisms generating the unique morphology of M1-derived MGCs.

## Materials and Methods

### Cell lines, chemicals and antibodies

The murine RAW 264.7 macrophage cell line was obtained from the American Type Culture Collection (Manassas, VA) and maintained at 37°C supplied with 5% CO_2_, in DMEM supplemented with 10% heat-inactivated FBS. For MGC differentiation, AMEM was used instead of DMEM.

LPS, IFNγ, taxol and cytochalasin D were purchased from Sigma-Aldrich Inc. (Oakville, ON). Mouse IL-4 was purchased from PeproTech (Dollard des Ormeaux, QC). Wiscostatin was purchased from Enzo Life Sciences (Brockville, ON). Blebbistatin was purchased from Toronto Research Chemicals (Toronto, ON). M-CSF and RANKL (for BMDM analysis) were obtained from R&D Systems (Minneapolis, MN). For western blotting, rabbit anti-iNOS and rabbit anti-GAPDH-HRP antibodies were purchased from Cell Signaling Technology (Whitby, ON). Rabbit anti-MMP-9 and rabbit anti-cathepsin K antibodies were purchased from Abcam (Toronto, ON). For immunostaining, Alexa-Fluor phalloidin (Invitrogen, Burlington, ON) was used to stain the actin cytoskeleton while rabbit and mouse α-tubulin (Sigma, Oakville, ON) and mouse acetylated α-tubulin (Sigma, Oakville, ON) were used to stain the microtubule network. Mouse GM130 antibody was purchased from BD Transduction Laboratories (San Jose, CA).

### GST-RANKL production and purification

BL21 E. *coli* transformed with a pGEX-GST-hRANKL vector (a gift from Morris Manolson, Dentistry, University of Toronto) was grown from a starter culture in LB broth containing ampicillin at 37°C (230 rpm) until an OD_690_ of 0.6–0.8. Cultures were induced with 2 mM IPTG at room temperature with gentle shaking overnight. Cultures were then centrifuged (4°C at 6000 rpm for 15 min), resuspended (1X PBS containing 150 mM NaCl, 1 mM EDTA, 1∶100 BPI, 1∶1 lysozyme, 1% Triton X-100) and sonicated at medium intensity. Sonicated cells were incubated with 1∶1000 DNaseI for 20 minutes prior to centrifugation (4°C at 17000 rpm for 15 min). Supernatant was incubated with Glutathione Sepharose 4B High Performance beads (GE Healthcare) for 30 min at 4°C on a rotator. Beads were transferred to empty Bio-Rad chromatography columns and were washed four times with chilled 1X PBS before elution buffer (10 mM Glutathione in Tris-HCl 50 mM, pH 8.0) was added for 20 min at room temperature. Three elutions were performed. Relative concentrations were determined using a Bradford Assay.

### Generation of multinucleated giant cells

**Figure 1:**
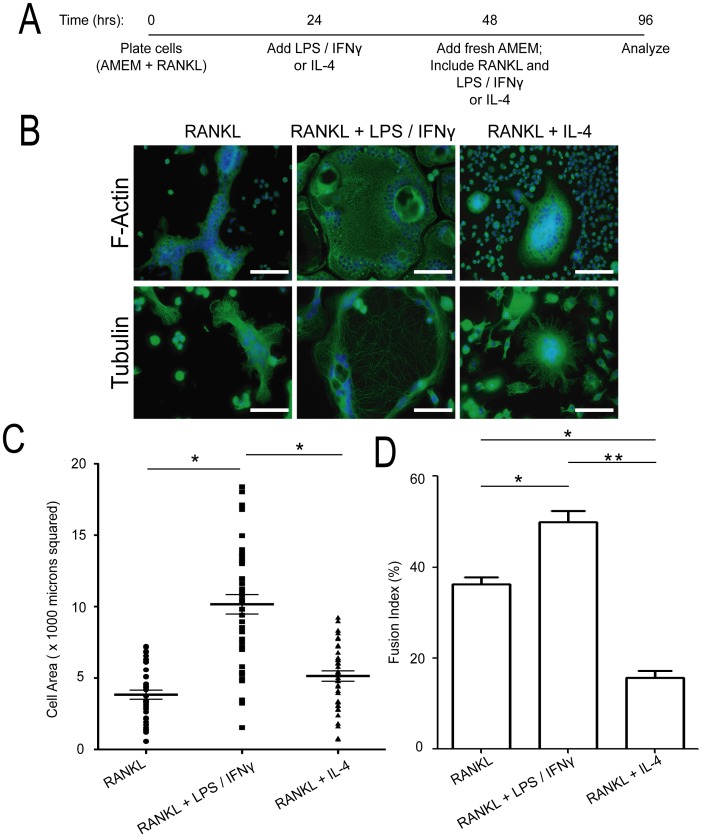
Characterization of the multinucleated giant cells generated by RANKL, RANKL+ LPS/IFNγ, and by RANKL+ IL-4. (A) Schematic of protocol used to generate the various multinucleated giant cells (MGCs). RAW 264.7 cells were plated on 6-well plates, with or without glass coverslips (160 000–180 000 cells/well). Cells were plated in AMEM with 25 ng/ml RANKL. The next day (day 1; 24 hrs later), LPS and IFNγ (0.1 µg/ml and 100 U/ml, respectively) or IL-4 (10 U/ml) were added. On day 2 (48 hrs later), the media was changed, and RANKL, LPS, IFNγ, or IL-4 were added. Experiments were terminated on day 4. (B) Representative images of day 4 MGCs, analyzed by immunofluorescence microscopy for DNA (blue) and F-actin (green; top panel) and α-tubulin (green; bottom panel). Scale bars represent 50 microns. (C) Quantification of cell size (area) of the various MGCs (mean ± standard deviation). MGCs were generated four independent times and between 40–50 MGCs were measured at each time point. * indicates *p*<0.0001 (*ANOVA*). (D) Quantification of fusion index of the MGCs generated in (C). * indicates *p*<0.01, ** indicates *p*<0.0005 (*ANOVA*).

Previous studies of osteoclastogenesis and multinucleated giant cell formation have used various protocols resulting in sometimes conflicting results. Protocol variations include the amount, timing, and duration of LPS, IFNγ, or IL-4 treatment, the presence or absence of RANKL in the media, the precursor cell line, the substrate on which the cells are initially plated, and the assessments used to determine osteoclast-likeness. In order to maintain consistency, the following protocol was used (Fig. 1A). In brief, RAW 264.7 murine macrophages were initially plated on 6-well plates (with or without glass coverslips) or on Corning Osteo Assay Surface (24-well) Plates (Corning; Corning, NY) in AMEM media containing 25 ng/ml RANKL. Following 24 hours (24 hours post-plating; Day 1), either LPS and IFNγ (0.1 µg/ml and 100 U/ml, respectively) or IL-4 (10 U/ml) was added. The next day (48 hours post-plating; Day 2), the media was replaced with fresh media containing RANKL and LPS/IFNγ or IL-4. On Day 4 (96 hours post-plating), the experiment was terminated (or drugs were added for a specific time and then the experiment was terminated). The quantity of LPS, IFNγ and IL-4 added were based on what is commonly used to activate macrophages [15], [20]. At the end of the experiment, the generated MGCs were assessed, via microscopy and protein analysis, for osteoclast-likeness (TRAP staining, phagocytic ability, and cathepsin K and MMP-9 expression).

To generate multinucleated giant cells from primary cells, primary precursor cells were isolated from long bones (femur and tibia) of mice. The cells were maintained in a modified essential medium (A-MEM), supplemented with 10% fetal bovine serum, 1% antibiotic-antimycotic solution, and M-CSF (25 ng/ml). BMDMs were generated by incubating adherent cells in M-CSF (100 ng/ml) and commercial RANKL (100 ng/ml) for five days, and maintained in 5% CO_2_. MGCs were generated following the five days by adding either LPS and IFNγ (0.1 µg/ml and 100 U/ml, respectively), or IL-4 (10 U/ml), for one week (with media changes every other day). Assays were terminated after the 7^th^ day.

### Immunofluorescence

Multinucleated giant cells (MGCs) were generated on coverslips in 6-well tissue culture plates. At the end of the protocol, cells were fixed with 4% PFA, permeabilized with 0.1% Triton X-100, and blocked with 5% FBS for 1 hour. Cells were incubated with primary and secondary antibodies for 1 hour (room temperature), followed by DAPI staining for 10 minutes. Coverslips were then mounted on glass slides and analyzed the next day with an inverted Zeiss AxioObserver.Z1 epifluorescence microscope using the AxioVision software (Zeiss, Thornwood, NY). Live cell imaging experiments were conducted at 37°C and 5% CO_2_, using an Incubator XL-S1 with TempModule S1, CO_2_ module S1, heating insert P S1 and heating device humidity S1 mounted on AxioObserver Z1.

### TRAP staining and fusion index calculation

Multinucleated giant cells (MGCs), generated by stimulated RAW 264.7 cells on coverslips in 24-well tissue culture dishes, were stained for tartarate-resistant acid phosphatase using the Acid Phosphatase (TRAP), Leukocyte Kit (Sigma, Oakville, ON). Briefly, cells were fixed for one minute with the fixative solution at room temperature, followed by staining with the staining solution for 1 hour at 37°C. Following water washes, coverslips were air dried and analyzed microscopically using a Zeiss Axioplan 2 epifluorescent microscope with a black and white AxioCam HRm and colour AxioCam HRc. MGCs generated by RAW 264.7 cells on Osteo Assay Surfaces were stained stained similarly with the exception that cells were fixed with 4% PFA instead of the suggested fixative solution.

The fusion index was calculated by dividing the number of nuclei in MGCs by the total number of nuclei in the field of view. The index was averaged over four independent experiments, with 40–50 MGCs analyzed per experiment.

### FcγR-mediated phagocytosis

MGCs were generated on coverslips in 6-well tissue culture plates. On the day of the assay, sheep RBCs (sRBCs; MP Biomedicals, Santa Ana, CA) were opsonized with rabbit anti-sheep RBC IgG (MP Biomedicals, Santa Ana, CA) for 1 hour at room temperature. Following three PBS washes, the MGCs were challenged with IgG-sRBCs for 10 minutes (binding). After removal of unbound IgG-sRBCs via PBS washes, the cells were incubated at 37°C for 10 minutes (particle internalization). Following lysis of external, bound IgG-sRBCs (20 second water wash) and three PBS washes, cells were fixed with 100% methanol at −20°C for 10 minutes. Cells were then immunostained (actin, IgG-sRBCs and nuclei) and analyzed by immunofluorescence microscopy.

### Bone resorption assays

RAW 264.7 stimulated cells were allowed to form multinucleated giant cells on Osteo Assay Surface plates. Following the differentation protocol, cells were removed using a 2% hypochlorite solution for 5 minutes, rinsed with distilled water, and air-dried. For staining, plates were treated in darkness, at room temperature, with 100 µl/well of a 2.5% (w/v) silver nitrate solution for 20 minutes. Wells were then aspirated and washed for 5 minutes with distilled water. Wells were again aspirated, and incubated (room temperature; 5 minutes) in 100 µl/well of a 5% (w/v) sodium carbonate in 10% formalin solution. Plates were then washed twice with PBS, rinsed thrice with distilled water, and dried in a 50°C incubator for an hour. Plates were then imaged with a Zeiss Axioplan 2 epifluorescent microscope with a black and white AxioCam HRm and colour AxioCam HRc.

### Western blotting and antibodies

During the differentiation protocol, total cell lysates were collected by scraping cells in radioimmunoprecipitation assay (RIPA) buffer (50 mM Tris-Cl, pH 7.4, 1% Triton X-100, 1% sodium deoxycholate, 0.1% SDS, 1 mM EDTA, pH 7.0, 150 mM NaCl, 1% aprotonin, 1 mg/ml leupeptin, 50 mM NaF, 1 mM Na_3_VO_4_, 10 µg/ml pepstatin in ethanol, and 1 mM phenylmethylsulfonyl fluoride in Me_2_SO) containing protease and phosphatase inhibitors. Following protein quantification, equal amounts (20 µg) were loaded on 8% or 15% SDS-PAGE gels, transferred onto nitrocellulose membranes, and blocked with 5% milk for 1 hour. Blots were incubated with primary antibodies overnight at 4°C and with secondary antibodies for 1 hour.

## Results

### Multinucleated giant cell cytoskeleton and morphology

Using our RAW 264.7 macrophages differentiation protocol (Fig. 1A), we first examined the cytoskeletal network in differentially stimulated cells (Fig. 1B). RANKL-treated MGCs had pleomorphic shapes with frequent F-actin and microtubule-rich cellular protrusions (Fig. 1B). They varied in size (area) from ∼500 µm^2^ to ∼7000 µm^2^ and contained between 4 to greater than 30 nuclei that were found through the entirety of the MGC (Fig. 1C). In contrast, RANKL+ LPS/IFNγ-treated MGCs showed a distinct morphology. These cells were epitheliod in shape, ranging from ∼500 µm^2^ to >18000 µm^2^ (Fig. 1B,C). In fact, there was a subpopulation of these cells that were too big to fit into the field of view and thus, not counted in the analysis. These cells contained between 5–20 nuclei, which were arranged near the cell periphery and around vacuole-like structures. While the F-actin network showed no substantial staining variation throughout the cell's entirety, the microtubule network showed dense cortical staining with sparse microtubules throughout the rest of the cytosol (Fig. 1B). RANKL+ IL-4-treated MGCs had a characteristic oval or circular shape, with centrally-positioned nuclei (between 5–20) (Fig. 1B). Actin and microtubule staining showed a more concentrated region of these networks at the cell centre, particularly the microtubules, with less distribution around the cell periphery. The sizes of these MGCs ranged from ∼500 µm^2^ to ∼9000 µm^2^ (Fig. 1C). In order to determine whether LPS/IFNγ or IL-4 had any effect on the ability of RANKL-treated RAW 264.7 macrophages to fuse, we quantified the fusion index across the three MGC types (Fig. 1D). RANKL-treated MGCs had a fusion index of ∼37%, while RANKL+ LPS/IFNγ and RANKL+ IL-4 MGCs had a fusion index of ∼50% and 15%, respectively.

To verify whether these observations were limited to RAW 264.7 cells, we generated MGCs from bone marrow derived macrophages (BMDMs) using our modified protocol (Fig. S1A). Similar to observations in RAW 264.7 cells, MGCs derived from BMDMs showed similar morphological characteristics when treated with RANKL, RANKL+ LPS/IFNγ, or RANKL+ IL-4 (Fig. S1B). When cell sizes were compared, RANKL+ LPS/IFNγ-treated MGCs were once again significantly larger that RANKL alone or RANKL+ IL-4-treated MGCs (Fig. S1C). However, unlike in RAW 264.7-derived MGCs, there was no significant difference in the sizes of RANKL-treated and RANKL+ IL-4 MGCs. A comparison of the fusion index also showed some different trends. While BMDM-MGCs formed due to RANKL+ LPS/IFNγ treatment showed similar fusion index values to those seen with RAW 264.7-MGCs, RANKL alone treatment and RANKL+ IL-4 MGCs were different: RANKL alone treated BMDM-MGCs had a fusion index of ∼20% (compared to ∼40% in RAW 264.7-MGCs) whereas RANKL+ IL-4 treated BMDM-MGCs has an index of ∼40% (compared to ∼20%).

**Figure 2:**
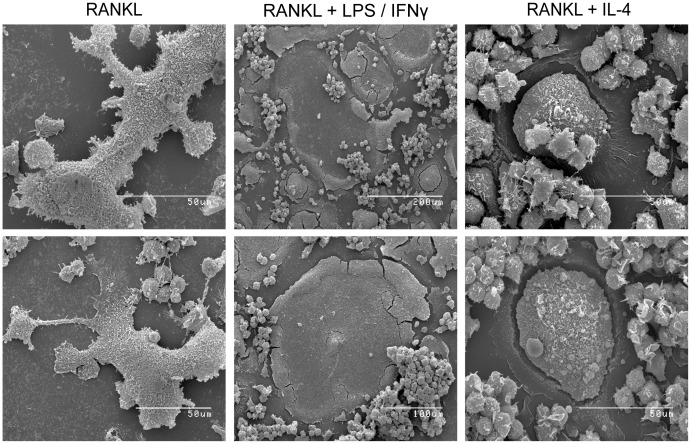
Characterization of the ultrastructural differences of the various MGCs. Representative scanning electron microscopy images of the generated day 4 MGCs. Scale bars represent 50(middle-top and middle-bottom, respectively).

To further analyze the morphology of the various generated MGCs at an ultrastructural level, scanning electron microscope images were taken (Fig. 2). Once again, RANKL-treated MGCs exhibited no defined morphology, and heterogenous shapes and cellular protrusions were observed. However, RANKL+ LPS/IFNγ-treated MGCs had a characteristic indentation throughout most of the cell body that was surrounded by a visibly thicker border area (Fig. 2). In some of these cells, there were upward protrusions in the indentation. RANKL+ IL-4-treated MGCs showed a consistent oval/circular morphology and, as the actin and microtubule staining suggested, the centre of the cell was visibly thicker than the periphery (Fig. 2).

### Osteoclast – likeness: functional assays

**Figure 3:**
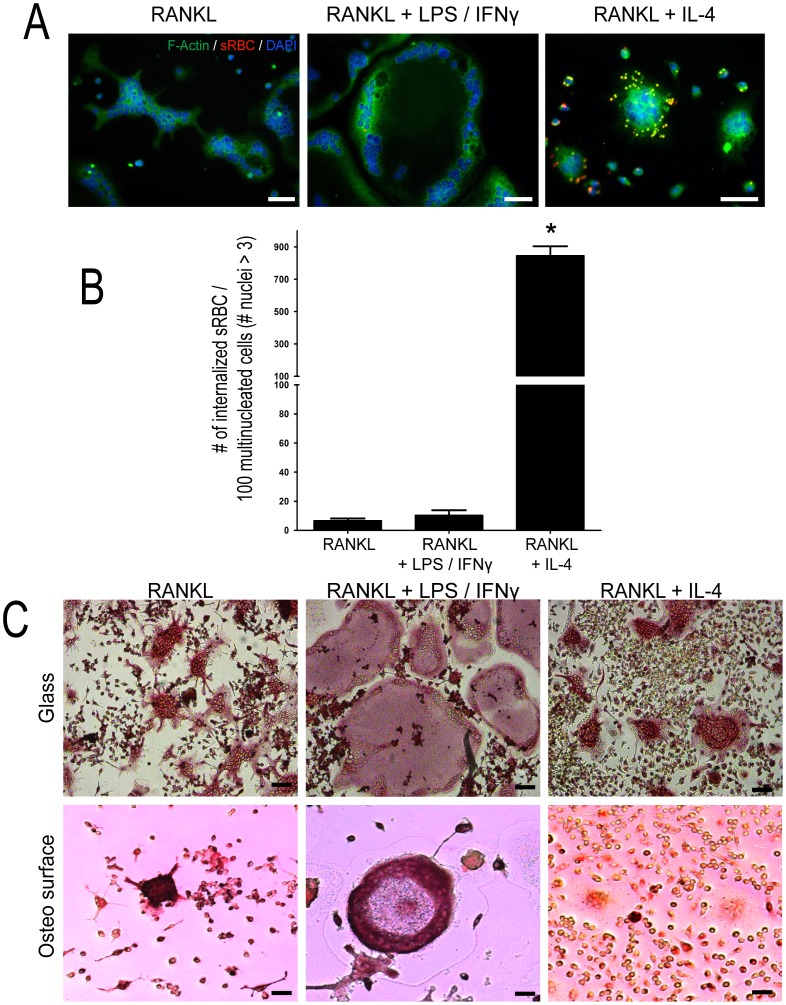
Functional assays of generated MGCs. Day 4 MGCs were assessed on their ability to undergo phagocytosis of IgG-opsonized sheep red blood cells (IgG-sRBCs) and on their ability to produce TRAP. (A) Representative images of cells after phagocytosis assay (see Methods). IgG-sRBCs are in red, F-actin in green, and DNA in blue. (B) Quantification of internalized IgG-sRBCs per 100 MGCs (mean ± standard deviation). Quantification was done on three independent experiments, with between 100–150 MGCs counted per experiment. * indicates *p*<0.001 (*ANOVA*). (C) Representative images of day 4 MGCs stained for TRAP. Top panel: RAW 264.7 macrophages on glass substrate. Bottom panel: RAW 264.7 macrophages on Osteo Assay Surface. Scale bars represent 50 microns.

A key component of macrophage function is their ability to carry out phagocytosis [21]. One type of phagocytosis employed by these cells is through Fcgamma (Fcγ) receptors, which engage IgG-opsonized targets (or IgG-sensitized red blood cells). Osteoclasts characteristically lose their phagocytic ability throughout differentiation, which is attributed to reduced Fcγ receptor display [22]. We thus determined the ability of the various induced MGCs to internalize IgG-opsonized sheep red blood cells (IgG-sRBCs). Of the three different MGC types, only RANKL+ IL-4-treated MGCs showed significant ability to internalize IgG-sRBCs (Fig. 3A,B; *p*<0.0001). While there were some RANKL− and RANKL+ LPS/IFNγ-treated cells that did have internalized IgG-sRBCs, the frequency was very rare (∼5–10 internalized IgG-sRBCs per 100 MGCs) (Fig. 3A,B). These observations suggested that RANKL-treatment alone and RANKL+ LPS/IFNγ-treatment either decreased Fcγ receptor expression post macrophage fusion, or significantly decreased internalization post fusion, and IL-4 treatment either prevented this loss, or activated their expression.

RANKL treatment of RAW 264.7 cells, plated on bone slices, bone-mimetics or glass slides, leads to osteoclast formation [23], [24], [25], [26], [27]. Two traditional assays used to test for osteoclast presence are the tartarate resistant acid phosphatase (TRAP) assay and bone resorption assays [7]. RAW 264.7 cells were plated and differentiated on glass coverslips and Osteo surfaces and stained for TRAP at the end of the protocol. As expected, on glass substrate, RANKL-treated cells stained positive for TRAP, with intense reddish brown staining throughout the cytoplasm (Fig. 3C). Similarly, RANKL+ IL-4-treated MGCs also stained positive for TRAP. Interestingly, RANKL+ LPS/IFNγ-treated MGCs had the opposite distribution of TRAP, compared to RANKL and RANKL+ IL-4-treated MGCs, with more intense TRAP staining at the cell borders and diffuse staining at the cell centre. Thus all induced MGC cell types were positive for TRAP with notable intensity and localization differences. While similar trends were observed on Osteo surfaces, there were some notable differences. RANKL-treated MGCs and RANKL+ LPS/IFNγ-treated MGCs showed strong TRAP staining, however RANKL+ IL-4-treated MGCs had substantially weaker staining profiles when compared to RANKL-alone cells (Fig. 3C).

**Figure 4:**
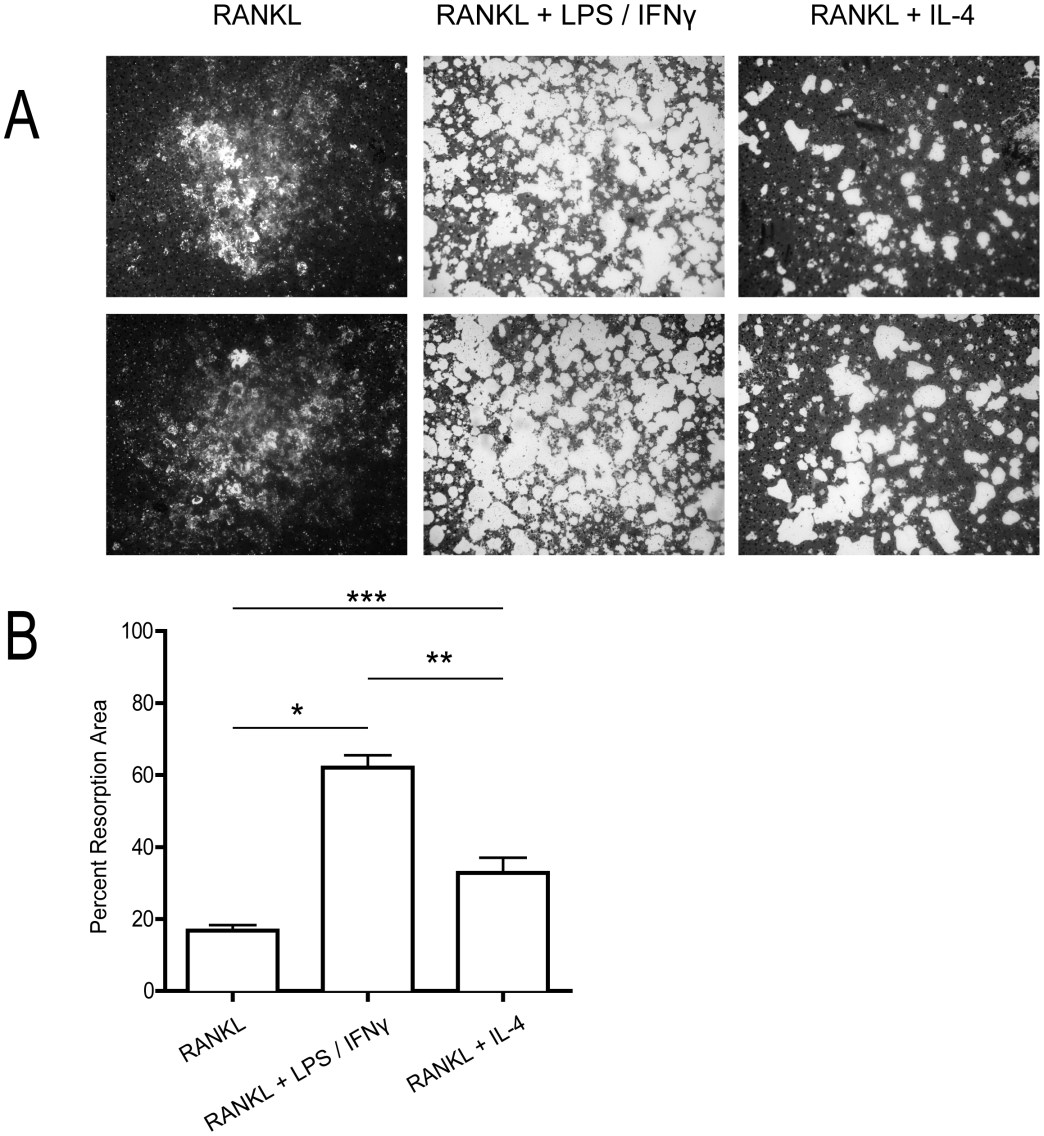
Resorption ability of generated MGCs. RAW 264.7 macrophages were plated and differentiated on Osteo Assay Surface plates. At the end of the assay, the MGCs were assessed on their ability to resorb the substrate throughout the protocol. (A) Two representative images of resorption pits by the various treated MGCs. (B) Quantification of resorption area (mean ± standard deviation of 3 independent assays) as determined by percentage resorbed versus total area. * indicates *p*<0.001, ** indicates *p*<0.01, and *** indicates *p*<0.05 (*ANOVA*).

In order to identify whether the MGCs generated on non-glass substrates had bone resorption capabilities, RAW 264.7 cells were plated and differentiated on Osteo surfaces. Results showed that all three MGC-types were able to resorb bone (Fig. 4). RANKL+ LPS/IFNγ-treated MGCs showed the greatest resorption ability, likely due to their size difference, followed by RANKL+ IL-4-treated cells, and finally RANKL-alone cells. This data, coupled with TRAP analysis of the MGCs suggest that while the various MGC may have different morphologies, they do share similar functional characteristics as OCs.

### Osteoclast – likeness: protein markers

**Figure 5:**
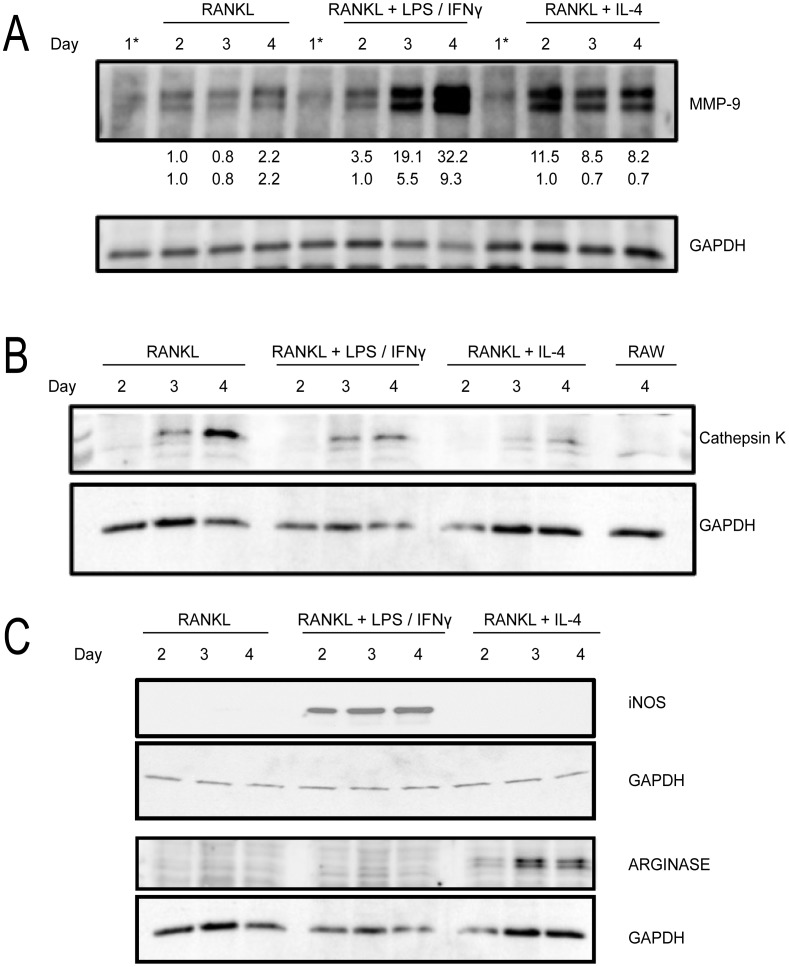
Biochemical characterization of the osteoclast-likeness of the various MGCs. (A) Cell lysates were taken at indicated time points during the differentiation protocol and analyzed for MMP-9 by Western blotting. 1* indicates lysates from RAW 264.7 cells prior to LPS/IFNγ or IL-4 addition. Numbers below MMP-9 blot represent densitometry readings for MMP-9 expression with 1.0 being day 2, RANKL-treated cells. (B) Cell lysates were taken from day 2 – day 4 during the differentiation protocol and analyzed for Cathepsin K expression. The last lane is the cell lysate from day 4 untreated RAW 264.7 cells. (C) Same as (B) but blots were analyzed for Arginase and iNOS (arrow). GAPDH was used as a loading control. Blots are representative of three independent experiments.

Other means to characterize osteoclast activity include probing for characteristic osteoclast protein biomarkers. First, we probed for MMP-9, a protease secreted by functional OCs [13], [14] (Fig. 5A). RANKL-treated cells showed a gradual increase in MMP-9 expression, showing a ∼2-fold increase between day 2 and day 4. RANKL+ LPS/IFNγ-treated cells also showed a similar trend, although the MMP-9 levels were more significantly enhanced at days 3 and 4 (Fig. 5A). In these cells, there was a ∼10-fold increase in MMP-9 expression between day 2 and day 4. In contrast, RANKL+ IL-4-treated cells showed an initial pronounced increase in MMP-9 expression between days 1 and 2 which then persisted throughout the time window of analysis (Fig. 5A). Thus, based on MMP-9 expression, RANKL and RANKL+ LPS/IFNγ showed similar MMP-9 temporal trends, albeit of different magnitudes.

Another characteristic osteoclast marker is the cathepsin K (CK) protease [13], [14], [15]. RANKL-treated cells showed a gradual increase in CK expression, with protein levels detected around day 3. RANKL+ LPS/IFNγ-treated cells also showed increasing CK expression, but levels were lower than RANKL-treated cells (Fig. 5B). In contrast, CK expression in untreated and IL-4-treated cells was very low (Fig. 5B).

### Multinucleated giant cells and macrophage activation markers

Traditionally, LPS/IFNγ and IL-4 are used to activate macrophages [15], [20]. LPS/IFNγ treatment “classically” activates macrophages while IL-4 is one way to “alternatively” activate macrophages. Each type of activation induces the production of certain protein biomarkers in the cell. We next wanted to determine if exposure to LPS/IFNγ or IL-4 influenced macrophage activation of RANKL-treated cells. RANKL-treated cells showed no detectable expression of iNOS nor arginase, known classical and IL-4-induced alternative activation markers, respectively (Fig. 5C) [28]. Interestingly, RANKL+ LPS/IFNγ-treated cells were positive for iNOS and negative for arginase while RANKL+ IL-4-treated cells were positive for arginase and negative for iNOS (Fig. 5C). This suggests that pre- or continued stimulation of macrophages with RANKL is modulated by classical or alternatively activation signals.

### Characterization of RANKL/LPS/IFNγ – induced MGCs

**Figure 6:**
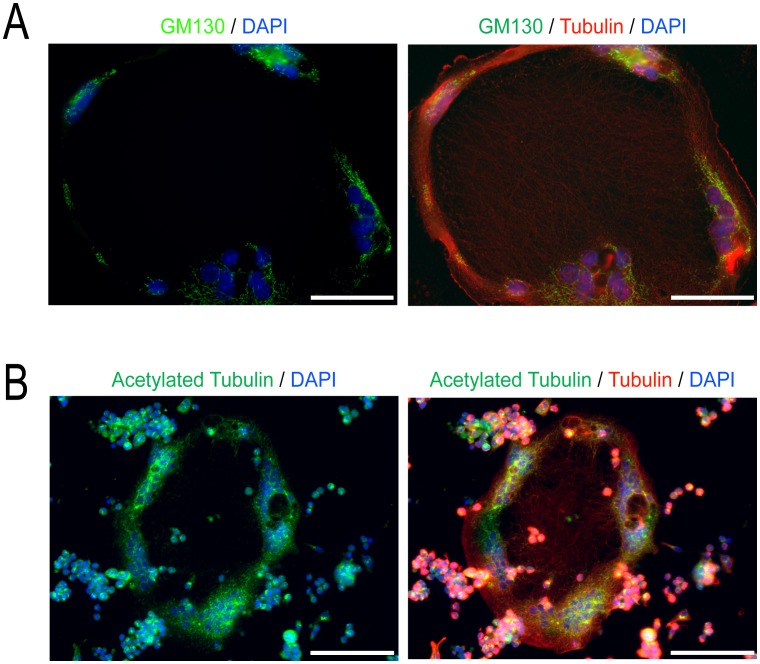
Golgi and stable microtubules are found at the periphery of RANKL+ LPS/IFNγ induced MGCs. (A) Representative images of day 4 MGCs treated with LPS/IFNγ and stained for GM130, a Golgi marker (green), α-tubulin (red), and nuclei (blue). (B) Representative images of day 4 MGCs treated with LPS/IFNγ stained for acetylated α-tubulin (green), α-tubulin (red), and DNA (blue). Images shown are representative of three independent experiments. Scale bars represent 50 microns.

To better understand how macrophage activation influences MGC formation, we next took a closer look at the nature of MGCs that were modulated by RANKL+ LPS/IFNγ. As described earlier, these were extremely large cells with a thin, compressed cell body. We examined the distribution of organelles in these cells, focusing on Golgi which were detected using antibodies against GM130 [29]. Multiple discrete Golgi were observed predominantly in the thicker, peripheral region of the cell, where the nuclei were located (Fig. 6A). As Golgi frequently localize towards the microtubule organizing centres (MTOCs) which are enriched with stable microtubules, we immunostained cells for acetylated tubulin, a marker of stable microtubules [30]. Acetylated microtubules were prominent encircling the peripheral cytoplasm, with only a few sparse microtubules penetrating the depressed cell interior (Fig. 6B).

Our imaging data suggested that RANKL+ LPS/IFNγ MGCs relied on the cytoskeleton to sequester the bulk of the cytoplasm towards the cell periphery. This peripheral distribution could be due to the selective microtubule trafficking of cytoplasm to the cell edge resulting in collapse of the cell centre or from dorsal acto-myosin compressive forces at the cell centre pushing the cellular contents outwards. Both scenarios would explain the indented central region of RANKL+ LPS/IFNγ MGCs observed by SEM in Fig. 2. To discriminate between compressive and pulling forces, we treated cells with specific cytoskeletal disrupting agents to release these forces.

**Figure 7:**
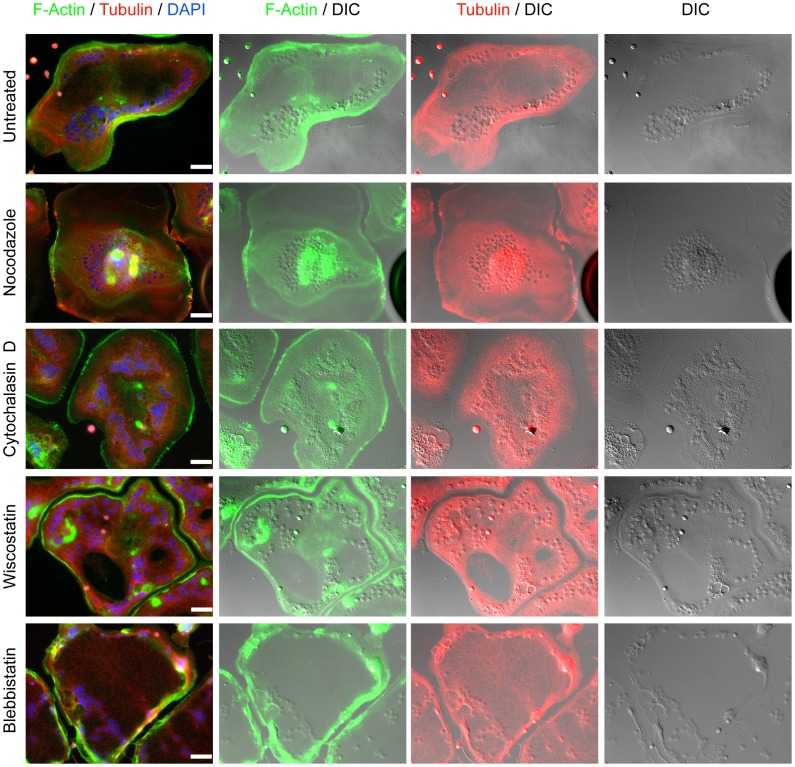
The effects of actin cytoskeleton, microtubule network, and myosin II disruption on the morphology of LPS/IFNγ induced MGCs. Day 4 LPS/IFNγ-induced MGCs were incubated with Nocodazole (5 µM), Cytochalasin D (1 µM), Wiscostatin (10 µM), or Blebbistatin (50 µM), for 4 hours. Cells were then stained for F-actin (green), α-tubulin (red), and DNA (blue). Images shown are representative of three independent experiments. Scale bars represent 50 microns.

To disrupt microtubule trafficking of organelles, RANKL+ LPS/IFNγ-induced MGCs were incubated with nocodazole for 3–4 hours after the 96-hour differentiation protocol (Fig. 7). As expected, nocodazole treatment resulted in the depolymerization of the microtubule network, which had a pronounced effect on the cell morphology. The MGCs no longer had the central indentation surrounded by a thicker layer at the periphery (Fig. 7). In fact, they appeared more like the MGCs formed by RANKL and IL-4, albeit with an epitheliod shape and larger size. Furthermore, the nuclei in these cells were no longer limited to the cell periphery and instead, were often found towards the centre of the cell (Fig. 7). These results indicated that the microtubule network played an important role in regulating the shape of these MGCs and the localization of their cytoplasmic contents including nuclei.

To address whether acto-myosin events led to the phenotypic peripheral organization of the cytoplasm in RANKL+ LPS/IFNγ-induced MGCs, we first disrupted F-actin assembly using Cytochalasin D [31]. Cytochalasin D treatment of RANKL and LPS/IFNγ-mediated MGCs had a different effect than nocodazole treatment. Longer treatments of cytochalasin D (more than 4 hours) resulted in a slow centrally-directed drifting of the thick cytoplasmic ring progressively inwards. Concurrent with the border movement was nuclei movement to the cell center (Fig. 7; Video S1). Lastly, as time progressed, the entire centre of the cell became more elevated. Time-lapse images video showed almost wave-like motions heading towards the centre of the cell (Video S1). Concurrently with this cytoplasmic reorganization was increased microtubule penetration into the cell interior (Fig. 7).

F-actin was also disrupted using wiscostatin, which inhibits N-WASP activation of the Arp2/3 complex [32]. The addition of this drug had a different effect than cytochalasin D. While cytochalasin D caused outside-in cytoplasmic streaming, wiscostatin had no such directional effect. MGCs treated with this drug had, over the 3–4 hour period, showed regions in the central indentation that lifted/propelled upwards. Instead of a continuous, wave-like elevation of the indentation, some specific regions showed elevation whereas others did not. Moreover, in those regions that had elevations, nuclei were also found, along with stronger and denser microtubule staining. Thus, N-WASP-mediated Arp2/3 complex function played an additional role in maintaining the cell's unique morphology along with actin polymerization.

Lastly, we looked at the role of the F-actin myosin II motor on the morphology of RANKL+ LPS/IFNγ-treated MGCs. MGCs incubated with blebbistatin, an inhibitor of myosin II [33], showed no major differences compared to untreated cells. They still contained a central indentation, that contained a less dense microtubule network, and had nuclei positioned throughout the cell periphery (Fig. 7). The only noticeable difference was in the thickness of the peripheral border region. Blebbistatin treatment appeared to decrease the width of the border region, leading to nuclei being arranged one beside the other (Fig. 6, Video S2), as opposed to untreated control cells where nuclei could be found in groups along the periphery (Fig. 7, Video S3). Furthermore, time-lapse images of blebbistatin-treated cells showed rippling (from the centre of the cell to the periphery) against the border region (Video S2).

### Microtubule organizing centre and nuclei movement

**Figure 8:**
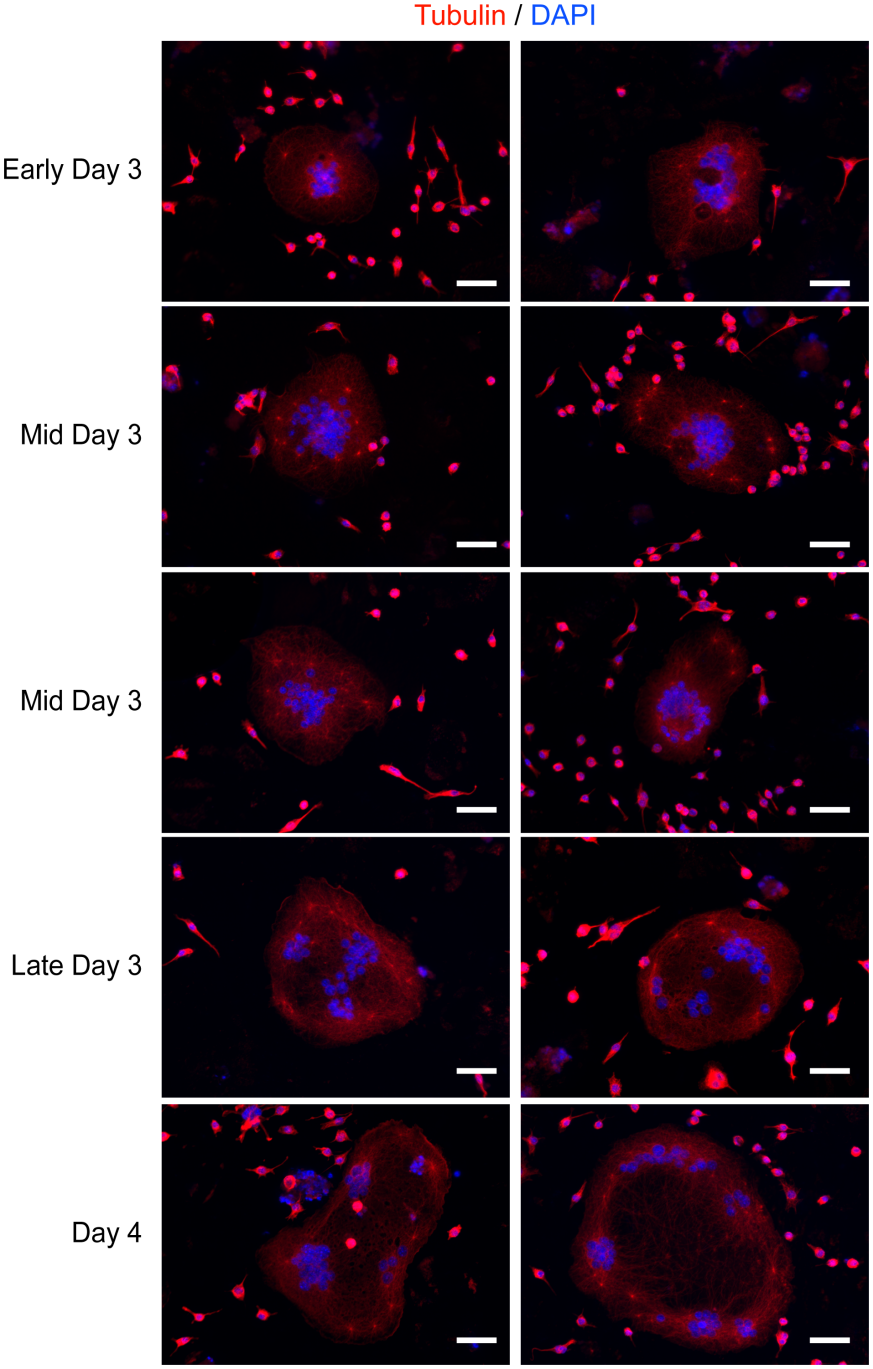
Microtubule-organizing centre movement during the differentiation of RANKL+ LPS/IFNγ-treated cells. Representative images of cells treated with RANKL and LPS/IFNγ from early Day 3 of the differentiation protocol until the end of experimentation (day 4). Cells were stained with tubulin (red) and DNA (DAPI). Images shown are representative of three independent experiments. Scale bars represent 50 microns.

Since the microtubule network seemed to play a role in the overall morphology of RANKL+ LPS/IFNγ-mediated MGCs, we looked at the microtubule organizing centre (MTOC) in these cells. In mononuclear cells, the movements of the MTOC and nucleus are closely coupled [34]. Furthermore, MTOCs are closely associated with the Golgi apparatus [35], [36]. We had observed in earlier experiments that α-tubulin staining of the various MGCs showed bright, punctate staining for MTOCs (data not shown). These were clearly visible and had microtubule strands emitting from them. Therefore, we decided to take fixed images of RANKL+ LPS/IFNγ-treated cells from early day 3 up to day 4 (Fig. 8). During our differentiation protocol, macrophage fusion began during late day 2 to early day 3. The nuclei in these cells were centralized, with MTOCs scattered between and/or around them (Fig. 8). At day 3, the MTOCs were found progressively further from the central core and closer to the cell periphery. A more noticeable movement of the nuclei towards the cell periphery occurred from late day 3 onwards (Fig. 8). Interestingly, the microtubule staining pattern followed a similar trend (Fig. 8). Up until late day 3, the staining profile was fairly even throughout the cell. Afterwards, a less dense microtubule staining was seen at the cell centre, again correlating the microtubule distribution with nuclear positioning.

## Discussion

The ability of cells of the monocyte/macrophage lineage to fuse and form multinucleated giant cells (MGCs) is well documented [2], [3], [4], [37], [38]. Fusion of these precursors *in vitro* and *in vivo* can lead to the formation of osteoclasts (OCs), Langhans giant cells (LGCs), foreign body giant cells (FBGCs), Toutons giant cells (TGCs), and giant cell tumours of the bone (GCTB) [2], [3], [4], [5], [37], [39]. However, irrespective of the manner by which they are generated, the role of these giant cells is similar: to help in the degradation of material (bone for OCs, bacteria for LGCs, foreign material for FBGCs, lipids for TGCs). Of particular interest for MGCs are how they are generated and whether their type can be altered. We chose to examine the fate of OCs once exposed to important macrophage modulators, namely the effects of classical (LPS/IFNγ) and alternative activation (IL-4) on OC-committed macrophages. While it is difficult to correctly determine what time interval of RANKL treatment is necessary to commit primary macrophages to the OC lineage, work done in murine macrophages (RAW 264.7) have shown that one needs at least 24 hours of RANKL treatment [40]. Furthermore, our RAW 264.7 and murine bone marrow derived macrophage cell differentiation protocols mimic physiological conditions wherein bacterial infections can occur while macrophages are being primed with RANKL. Here, we report that RANKL-treated macrophages can be differentiated into other types of MGCs if primed with activation signals prior to OC commitment. While classical activation (LPS/IFNγ) leads to the generation of LGC-type cells, alternative activation (IL-4) leads to the generation of cells similar to OCs with characteristics of FBGCs.

Our data suggests that the MGCs generated by RANKL-alone treatment are characteristic of OCs. Whether on bone or non-bone substrates, they exhibited morphological and/or protein biomarkers traditionally found in OCs. These results were then used to compare with those of the other MGCs that we generated. The MGCs generated by simultaneous RANKL and LPS/IFNγ had several similarities to normal OCs generated from RANKL alone. The RANKL and LPS/IFNγ stimulated cells had increasing MMP-9 and CK levels, were incapable of phagocytosis, were TRAP positive on glass and bone substrates, and were able to resorb bone substrates. However, the overall levels of the two proteases were different than observed in OCs. While MMP-9 expression was greater, CK expression was lower. Moreover, unlike the even, strong TRAP staining seen in normal OCs, the staining was limited to the cell periphery in RANKL and LPS/IFNγ-induced MGCs. The most striking feature of these MGCs was their size and morphology. MGCs from RANKL and LPS/IFNγ treatment were, on average, at least four times greater in area, and all showed a consistent, “indented pit surrounded by a border” phenotype. These observations suggested that LPS/IFNγ treatment led to cells similar to Langhans giant cells (LGCs) observed *in vivo* [2], [41], not OCs. Of particular interest in the MGCs formed by RANKL and LPS/IFNγ is the interplay between LPS and IFNγ during the differentiation process. Various studies have indicated a stimulatory and inhibitory role for both LPS and IFNγ, individually, to direct RANKL-pretreated macrophages towards the OC lineage [8], [42], [43]. What has not been elucidated is the combinatorial effect of chronic LPS and IFNγ exposure on RANKL-induced osteoclastogenesis. Our data suggests a shift from OCs towards LGC-type cells.

The role of IL-4 in OC formation has also been well characterized [9], [10], [44]. IL-4 has been linked with the suppression of osteoclastogenesis and osteoclast activity through the STAT6-dependent inhibition of NFκB [9], [44]. However in these studies, IL-4 was added to macrophages alongside RANKL, thereby preventing any effect of RANKL to potentially commit these cells to the osteoclast lineage. In one case, IL-4 was added to murine macrophages 48 hours post-RANKL treatment [9]. Interestingly, these cells did form giant cells but were classified as osteoclasts solely based on TRAP staining. On glass, the generated MGCs were TRAP positive, whereas on Osteo surfaces, the cells were only weakly positive. This suggests that cell-substrate interactions play a role in TRAP expression. While RANKL and IL-4 induced MGCs only weakly stained for TRAP on Osteo surfaces, we still found that were able to resorb bone. Thus TRAP staining alone may not fully reflect bone resorption potential of MGCs. Furthermore, unlike the previous study, we measured MMP-9 and CK protein expression and found differences between these MGCs and OCs. While these MGCs did express MMP-9, protein levels remained constant throughout the differentiation protocol; however, CK protein was barely detected. While we have no evidence regarding the purpose of MMP-9 secretion in IL-4 stimulated MGCs, we speculate that this may be related to the fusion process and the migratory capacity of foreign body giant cells [45], [46], [47]. We also observed an enhanced capacity for phagocytosis, a characteristic of FBGCs [38]. IL-4 (with no RANKL treatment) has been shown to lead to FBGC formation in several studies. However, FBGCs have been traditionally characterized as having no defined shape and no specific nuclei arrangement [3], [37], [38]. Furthermore, they have been shown to express CK [48]. Therefore, based on our data, MGCs generated by RANKL and IL-4 appear to have mixed biochemical and morphological characteristics of both OCs and FBGCs. More research is needed to properly define these cells and their function in immunological, developmental and disease processes.

Based on our *in vitro* data, we propose a model for Langhans-type giant cell formation as follows. The fusion of several RANKL+ LPS/IFNγ-treated mononuclear macrophages leads to giant cells with nuclei gathered together, and their MTOCs closely associated with and around them. Between 72 and 96 hours, the MTOCs of these cells undergo translocation to the cell periphery. Concomitant with their movement is that of the nuclei. It is clear from our data that the microtubule network plays a crucial role in the movement of these two elements. Since disruption of the microtubule network resulted in the inward movement of the nuclei, it is clear that microtubules are tethering the nuclei, and perhaps the MTOCs, to the cortex. While the cortical actin is a likely candidate, the addition of cytochalasin D and wiscostatin did not produce the drastic phenotypic changes as did nocodazole in the same time period, although these induced a clear morphological change. The prevention of actin polymerization by cytochalasin D led to the decrease in the central indentation area from the outside-in. This indicates that actin filament polymerization is important in maintaining the central depression closer to the cell periphery. Our findings indicate an inverse “lamellae-lamellipodia” orientation in RANKL+ LPS/IFNγ generated MGCs. Lamellipodia are the edge of motile cells and are thin, sheet-like membrane protrusions that are devoid of major organelles [49], [50]. More interior to the migrating front is the lamellae which has linear actin-bundles, cross-linked by myosin, in contrast to the lamellipodia that primarily consists of a F-actin meshwork [49], [50]. The actin filaments of lamellae are more stable and less dynamic than those of lamellipodia, and tend to resist compression. Within our generated Langhans-type giant cells, the interior seems to mimic lamellipodia, while the border region simulates lamellae. Thus, the addition of cytochalasin D preferentially affects filaments in this lamella – lamellipodia interface, resulting in an expanding lamella region.

This “inverse lamellae-lamellipodia” model is also supported by our wiscostatin data. Wiscostatin indirectly affects the Arp2/3 complex by inhibiting N-WASP activity [32]. Since Arp2/3 complexes are present at microfilament-microfilament junctions in lamellipodia [51], wiscostatin's effects would primarily be seen in the indentation side of the giant cells. In agreement, our data shows non-specific disruption of the indentation and not in the cell border/indentation interface. Interestingly, while both the disruption of Arp2/3 complex function and inhibition of actin polymerization reversed nuclei positioning/tethering towards the cell periphery, the nature of their action was different. The inhibition of actin polymerization by cytochalasin D loosened the cell border/indentation region, allowing for the simultaneous movement of nuclei inwards. This again suggests that there is a coordinated, condensed linear layer of stable actin bundles in that interface region between the border and indentation. Conversely, Arp2/3 complex inhibition produced rather random consequences. Only specific regions were “de-indented”, allowing for nuclear motion in those areas. Another consequence of our proposed model is the role of myosin II. Myosin II is generally found in the lamella of migrating cells instead of the lamellipodia [52]. The inhibition of myosin II in Langhans-type giant cells appeared to destabilize and reduce the thickness of the border of the giant cells, suggesting myosin II's presence in the cell border/indentation interface. These observations, coupled with those of the cytochalasin D experiment, suggest a role for condensed actin filament bundles and myosin II in the creation and maintenance of the border/indentation structure. While we have presented data as to the steps involved in the generation of these Langhans-type giant cells, a larger question still remains. How does RANKL+ LPS/IFNγ treatment lead to the formation of these MGCs? A possible answer to this question lies in studies showing the effects of LPS on interacting with, and modifying various microtubule associated proteins (MAPs), leading to the generation of stabilized and non-functional microtubules [53], [54], [55], [56]. Our lab has previously published reports characterizing the effects of LPS/IFNγ on microtubule stabilization and on putative LPS-MAP interactions in RAW 264.7 cells [57], [58], [59]. In the context of Langhans-type MGCs, it is possible that LPS/IFNγ treatment leads to the amplification of these modifications (phosphorylations) of specific MAPs, leading to the generation of unusually stable (acetylated) microtubules.

In conclusion, we have shown that OC commitment is not as firm as previously thought. Under certain circumstances, macrophages committed to the OC lineage can be altered to develop into other types of MGCs. Specifically, when faced with M1- or M2- inducing stimuli, pre-OCs can be reprogrammed to contribute to the heterogeneity of MGCs by modulating gene expression, cytoskeletal rearrangements, cell morphology and ultimately the physiological roles of these large and powerful cells. Furthermore, we provide insight into the roles of the actin and microtubule network in generating and maintaining the distinct phenotype of RANKL+ LPS/IFNγ-induced MGCs. This work may be helpful in the understanding of several bone and joint disorders that arise due to these large multinucleated cells.

## Supporting Information

Figure S1 **Characterization of BMDM-derived multinucleated giant cells generated by RANKL, RANKL+ LPS/IFNγ, and by RANKL+ IL-4.** (A) Schematic of protocol used to generate the various multinucleated giant cells (MGCs) from primary macrophages. Isolated precursor cells were plated on 6-well plates, with or without glass coverslips, in AMEM+ 100 ng/ml M-CSF and 100 ng/ml RANKL for 5 days to generate BMDMs. The next day, LPS and IFNγ (0.1 µg/ml and 100 U/ml, respectively) or IL-4 (10 U/ml) were added. Following 7 additional days, with media and cytokine replacement every other day, experiments were terminated. (B) Representative images of day 7 MGCs, analyzed by immunofluorescence microscopy for DNA (blue) and F-actin (green). Scale bars represent 50 microns. (C) Quantification of cell size (area) of the various MGCs (mean ± standard deviation). MGCs were generated four independent times and between 40–50 MGCs were measured at each time point. * indicates *p*<0.0001 (*ANOVA*). (D) Quantification of fusion index of the MGCs generated in (C). * indicates *p*<0.01, ** indicates *p*<0.0005 (*ANOVA*).
(EPS)

Video S1 **RANKL+ LPS/IFNγ-treated MGCs treated with cytochalasin D (1 µM) on day 4.** Images were acquired every 4 minutes with time-lapse epifluorescence imaging (AxioObserver, Carl Zeiss) for 21 hours and the playback speed was 30 fps.
(MOV)

Video S2 **RANKL+ LPS/IFNγ-treated MGCs treated with blebbistatin (50 µM) on day 4.** Images were acquired every 4 minutes with time-lapse epifluorescence imaging (AxioObserver, Carl Zeiss) for 21 hours and the playback speed was 30 fps.
(MOV)

Video S3 **Day 4 RANKL+ LPS/IFNγ-treated MGCs.** Images were acquired every 4 minutes with time-lapse epifluorescence imaging (AxioObserver, Carl Zeiss) for 21 hours and the playback speed was 30 fps.
(MOV)
